# Novel radiologic indices for stem type decision in total hip arthroplasty in patients with metaphyseo-diaphyseal mismatched Dorr A proximal femur

**DOI:** 10.1186/s12891-024-07223-5

**Published:** 2024-02-09

**Authors:** Han Jin Lee, Hong Seok Kim, Jeong Joon Yoo

**Affiliations:** 1 Department of Orthopaedic Surgery, Seoul National University College of Medicine, 101 Daehak-ro, Jongno-gu, Seoul, 03080 Korea; 2 Department of Orthopaedic Surgery, Hanil General Hospital, Seoul, Korea

**Keywords:** Total hip arthroplasty, Dorr A type, Stem selection, Metaphyseo-diaphyseal mismatch

## Abstract

**Background:**

In metaphyseo-diaphyseal (M-D) mismatched Dorr A femurs, it is difficult to achieve proper fixation with a type 1 stem. Proper interpretation of the geometry of the femur is integral at the preoperative stage in an M-D mismatched femur, but there has been a scarcity of studies on the radiologic indices. Therefore, we analyze the previous radiologic indices and suggest the novel ones for M-D mismatched femurs.

**Methods:**

Our study was a retrospective review of preoperative radiographs of patients who underwent total hip arthroplasty with the smallest type 1 stem or with type 3 C stem at a single institution from July 2014 to March 2022. A Type 3 C stem was used when the smallest type 1 stem failed to achieve metaphyseal fixation. One hundred twenty-six patients were categorized into two main groups. Canal-flare index, canal-calcar ratio, modified morphological cortical index, and two novel indices (lesser trochanter-to-distal ratio-α and -β [LDR-α and -β]) were assessed on preoperative pelvic radiographs.

**Results:**

Multivariate and ROC analysis demonstrated that high LDR-β (Exp[B]: 485.51, CI: 36.67-6427.97, *p* < 0.001) was associated with a more mismatched tendency group and had clinically acceptable discriminatory power (AUC: 0.765, CI: 0.675–0.855, *p* < 0.001) between the two cohorts.

**Conclusion:**

Correct assessment of preoperative femoral morphology would be fundamental in the selection of a suitable stem. The ratio based on 3 cm below the lesser trochanter of the femur seemed crucial. We recommend evaluating the newly described radiological index preoperatively in M-D mismatched Dorr A femur for planning precisely and selecting a proper stem.

**Supplementary Information:**

The online version contains supplementary material available at 10.1186/s12891-024-07223-5.

## Introduction

The number of cementless total hip arthroplasty (THA) is increasing [1]. As is known, type 1 stem, a proximally coated wedged stem, is based on the principle of a metaphyseal fixation, has been widely used, and exhibits excellent long-term follow-up results [2–5]. Nevertheless, the implantation of type 1 stem in a Dorr A femur may pose certain challenges. Dorr A femurs typically exhibit metaphyseo-diaphyseal (M-D) mismatch. Severe M-D mismatch, especially with abrupt narrowing in the M-D junctional area, may result in complications associated with the fixation of a type 1 stem in the diaphyseal or M-D junctional area. These complications include thigh pain, undersized stem selection, and failure of osteointegration [6–8], increasing the risk of early failure of implants [9–11]. To avoid the aforementioned complications, the surgeons should thoroughly assess the simple radiographs and prepare another type of stem such as a type 3 C stem, if necessary. Thus, in M-D mismatched femur, the proper preoperative selection of stem type is important, but there has been little study on the radiologic indices for measuring the severity of M-D mismatch precisely.

In the radiologic evaluation of femoral morphology, radiologic indices such as canal-flare index (CFI) [12], canal-calcar ratio (CCR) [13, 14], and morphological cortical index (MCI) [15] were widely used [16, 17]. However, the typical morphology of the proximal femur metaphysis, the important area for fixation of type 1 stem, could not be fully evaluated using previous radiologic indices. Previous studies reported that the femoral canal narrowed abruptly between the lesser trochanter (LT) and 5 cm below the LT in the Dorr A femur [18]. However, no proper indices represent the characteristics of this zone. Therefore, we defined novel radiologic indices using two points – 2 and 3 cm below the LT – to assess the degree of M-D mismatch of the Dorr A proximal femur.

The purpose of this study is to investigate the morphological differences of M-D mismatched proximal femur based on stem type differentiation by various radiological indices and to evaluate the efficacy of novel radiological indices in proper stem type selection in severe M-D mismatched Dorr A femur.

## Methods

### Study cohort

**Fig. 1:**
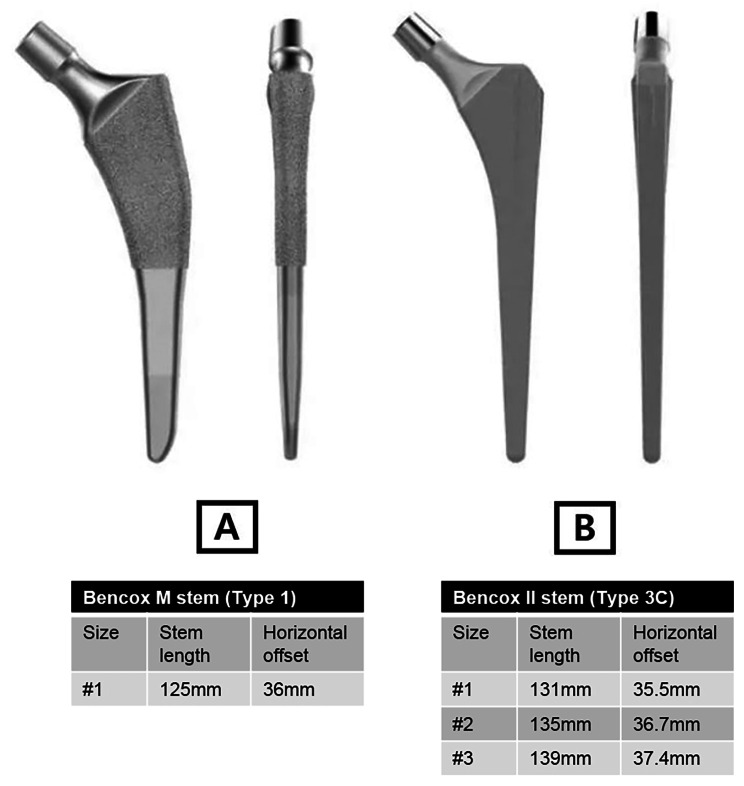
Morphological features of stems: (**A**) Bencox M stem (Type 1 stem); narrows medially-laterally, proximally coated, flat stem, thin in anterior-posterior plane (**B**) Bencox II stem (Type 3 C stem); rectangular cross section with four-point rotational support in metaphyseal-diaphyseal region

**Fig. 2:**
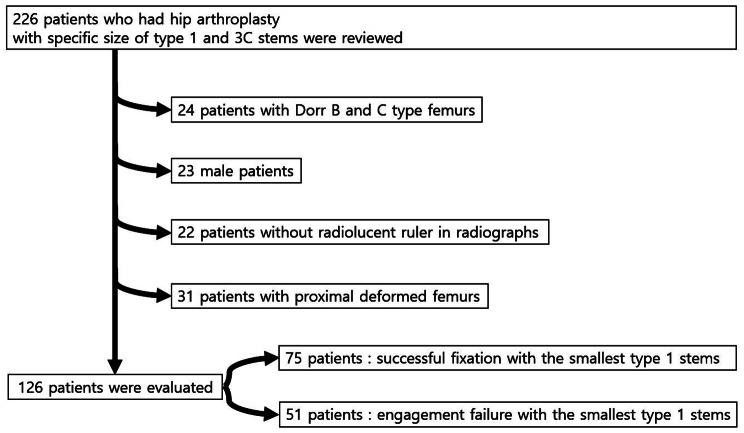
Study flowchart

This is a retrospective study, and the protocol had prior approval of the Institutional Review Board of Seoul National University Hospital (IRB No. H-2205-122-1327). Informed consent was waived as the study was performed retrospectively. From July 2014, we have preferentially used a cementless type 1 stem, Bencox M stem (Corentec, Cheonan, Korea). The smallest size of the Bencox M stem was tried first, and in cases where fixation was not achieved due to severe M-D mismatch, the Bencox II stem (Corentec), a type 3 C stem, was used instead (Fig. 1). Adequate stem fixation was assessed by intraoperative radiographs. Those who were successfully operated with type 1 stem were considered less M-D mismatched femur and grouped as the ‘Less Mismatched Tendency’ cohort. In contrast, patients whose intraoperative radiographs showed inadequate fixation of the smallest type 1 stem and ended up changing to the type 3 C stem, were grouped as the ‘More Mismatched Tendency’ cohort. Primary THAs performed with #1 size of Bencox M stem (stem length: 125 mm/horizontal offset: 36 mm) or #1, 2, or 3 sizes of Bencox II stem (#1; 131 mm/35.5 mm, #2; 135 mm/36.7 mm, #3; 139 mm/37.4 mm, respectively) from July 2014 to March 2022 were included for the evaluation. Dorr type was classified by the morphological characteristics of the proximal femur presented by Dorr et al. [19] and confirmed by the respective cut-off points of cortical index (CI) presented by Nakaya et al. [16]. CI is defined as the thickness of the femoral bone cortex at 10 cm below the LT divided by the external diameter at 10 cm below the LT. CI was evaluated on anteroposterior and lateral plain radiographs of the hip. The respective cut-off points of CI are > 0.58 on anteroposterior radiograph and > 0.45 on lateral radiograph. Exclusion criteria were (1) Dorr type B or C femurs; (2) no radiolucent ruler in preoperative radiographs; (3) male patients, and (4) severely deformed femurs (Fig. 2). Finally, 126 Dorr type A femurs of female patients were selected.

The preoperative diagnoses were osteonecrosis of the femoral head (42 cases), degenerative arthritis secondary to acetabular dysplasia (55 cases), secondary osteoarthritis related to other causes (15 cases), posttraumatic osteoarthritis (6 cases), rheumatoid arthritis (4 cases), and sequelae of Legg-Calves-Perthes disease (4 cases). (Table 1)

**Table 1:** Basic demographics of enrolled patients

	Less Mismatched Tendency [Type 1 stem] (*n* = 75)	More Mismatched Tendency [Type 3 C stem] (*n* = 51)	*p*-value
Age (yrs)	58.2 ± 13.2	53.3 ± 16.4	0.066
Diagnosis			0.248
ONFH	31 (41.3%)	11 (21.6%)	
Hip dysplasia	29 (38.7%)	26 (51.0%)	
Osteoarthritis	7 (9.3%)	8 (15.7%)	
Posttraumatic OA	4 (5.3%)	2 (3.9%)	
RA	2 (2.7%)	2 (3.9%)	
LCP seq	2 (2.7%)	2 (3.9%)	
Laterality			0.349
Right	46 (61.3%)	27 (52.9%)	
Left	29 (38.7%)	24 (47.1%)	
Body Mass Index (kg/m^2^)	25.4 ± 3.5	25.8 ± 4.6	0.583

### Operative procedure

**Fig. 3:**
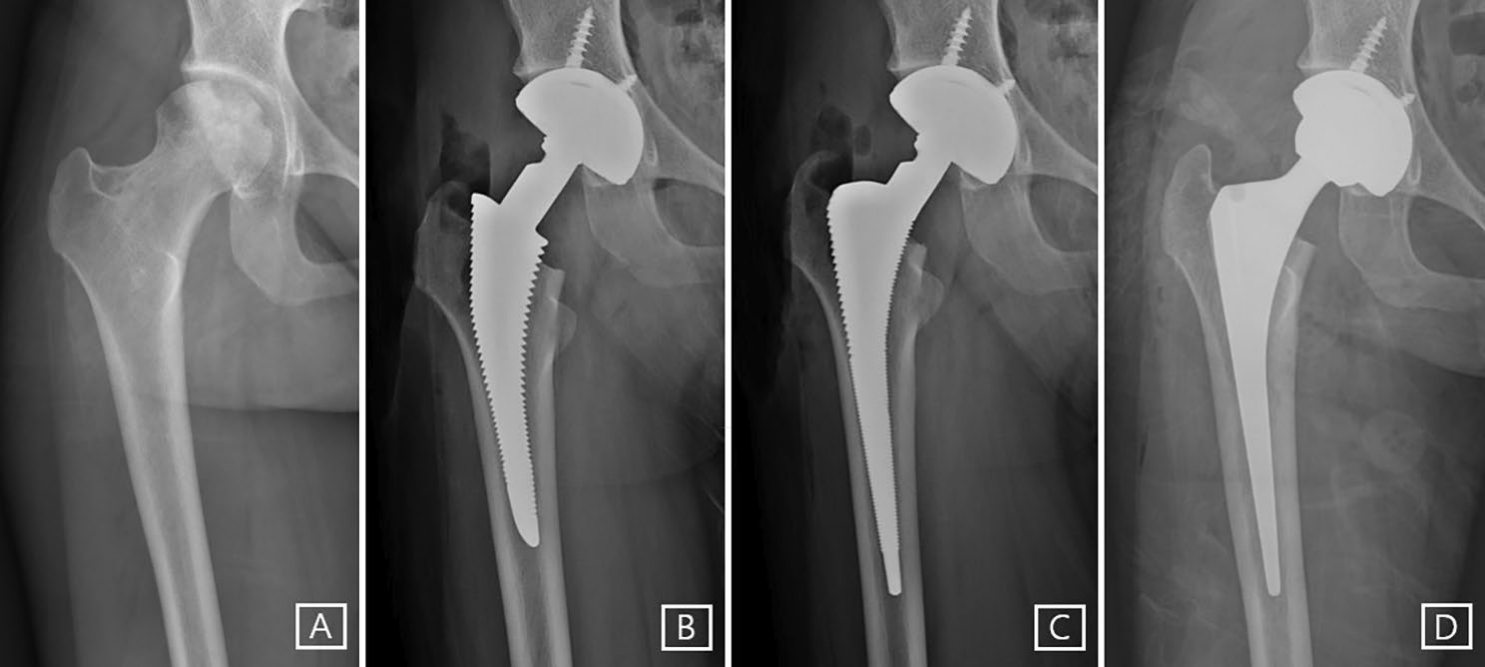
(**A**) A preoperative anteroposterior radiograph of 24-year-old woman with an osteonecrosis of the femoral head. She had a Dorr A femur, and we initially decided to use the smallest type 1 stem through preoperative templating. (**B**) In the intraoperative radiograph, the trial of the smallest type 1 stem could not be fully inserted and distal part of the stem was captured by narrow femoral canal due to severe metaphyseo-diaphyseal (M-D) mismatch. (**C**) In the consequent intraoperative radiograph, the trial of type 3 C stem was fully inserted and properly located. (**D**) The postoperative radiograph showed properly located and stably fixed implants

All index surgeries were performed by a single surgeon via modified direct lateral approach. The smallest type 1 stem was preferentially used. However, in femurs with severe M-D mismatch, the trial of smallest type 1 stem might not be properly placed in the metaphyseal area, which lead to additional preparation such as medullary curettage or extra-neck cutting. In cases where type 1 stem could not be properly positioned even with aforementioned procedure, type 3 C stem was selected. Also, if the gap between the trial of stem and the lateral or medial cortex of the host bone was visible after the trial insertion, the surgeon thought that there was a high risk of failure of osteointegration and changed it to type 3 C stem (Fig. 3).

### Radiographic assessments

Radiographic evaluation of femoral morphology was performed on preoperative anteroposterior hip radiographs. Two independent fellowship-trained orthopedic surgeons who were not involved in the surgery analyzed the radiographs. The measurements were repeated 6 weeks later.

CFI, CCR, and modified MCI were evaluated as well as novel radiologic indices. The modified MCI is defined as the internal diameter of the femur at the LT divided by the internal diameter of the femur at 7 cm below the LT, since MCI consists of the external diameter of the femur at the LT.

**Fig. 4:**
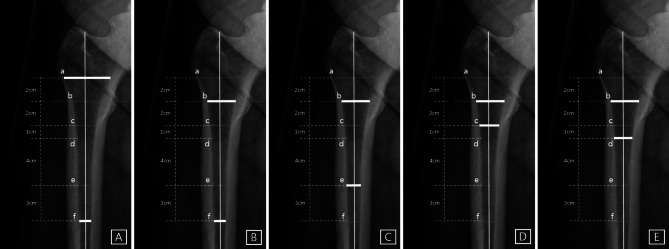
Radiographic parameters used to analyze proximal femoral morphology: (**A**) canal–flare index = a/f (**B**) canal–calcar ratio = f/b (**C**) modified morphological cortical index = b/e (**D**) LT-to-distal ratio-α = b/c (E) LT-to-distal ratio-β = b/d

Two novel radiologic indices were also used to analyze the radiographs: LT-to-distal ratio-α (LDR-α) is defined as the internal diameter at the LT divided by the internal diameter at 2 cm below the LT. LDR-β is defined as the internal diameter at the LT divided by the internal diameter at 3 cm below the LT (Fig. 4).

### Statistical analysis

Continuous variables are expressed as mean and standard deviation. Cross-tabulated data were compared using the chi-squared or Fisher’s exact test, and odds ratios with 95% confidence intervals were calculated. Student’s t-test was used for continuous variables. Inter- and intra-observer reliabilities of radiologic indices were evaluated using the intraclass-correlation coefficient (ICC). The definition of ICC values was as follows: excellent reliability (> 0.90), good reliability (0.75–0.90), moderate reliability (0.50–0.75), and poor reliability (< 0.50) [20]. A univariate analysis was performed on radiologic indices between the two cohorts, and a multivariate logistic regression analysis was analyzed. The multi-collinearity of variables was confirmed by the variance inflation factor. The area under the receiver operating characteristic (ROC) curve (AUC) of > 0.7 means a fair test with clinically acceptable discriminatory power [21, 22]. A *p*-value of < 0.05 was considered statistically significant. Statistical analyses were performed using IBM SPSS Statistics for Windows, version 26.0 (IBM Corp., Armonk, NY, USA).

## Results

The inter-observer and intra-observer agreement for each radiologic index measurement demonstrated excellent reliability, with values exceeding 0.90 (Supplementary Table 1). The mean CCR was significantly lower in the More Mismatched Tendency group (*p* < 0.001). The mean CFI and modified MCI were both significantly higher in the More Mismatched Tendency group (*p* < 0.001 and *p* < 0.001). The mean LDR-α and LDR-β were significantly higher in the More Mismatched Tendency group (*p* < 0.001 and *p* < 0.001) (Table 2).

**Table 2:** Preoperative radiologic parameters of the two cohorts

	Less Mismatched Tendency (Type 1 stem)	More Mismatched Tendency (Type 3 C stem)	*p*-value
Number	75	51	
Canal-calcar ratio (CCR)	0.40 ± 0.06	0.36 ± 0.05	< 0.001
Canal-flare index (CFI)	4.45 ± 0.60	4.85 ± 0.71	< 0.001
Modified morphological cortical index	2.27 ± 0.30	2.53 ± 0.30	< 0.001
LT-to-distal ratio-α (LDR-α)	1.50 ± 0.12	1.62 ± 0.16	< 0.001
LT-to-distal ratio-β (LDR-β)	1.74 ± 0.15	1.92 ± 0.20	< 0.001

The five variables identified in a univariate analysis were analyzed in the multivariate logistic regression model. There was no multi-collinearity among the five variables. A multivariate analysis demonstrated that Nagelkerke R^2^ was 0.292 and LDR-β was only predictive of severe M-D mismatch (OR = 485.51 [95% CI, 36.67-6427.97], *p* < 0.001).

**Fig. 5:**
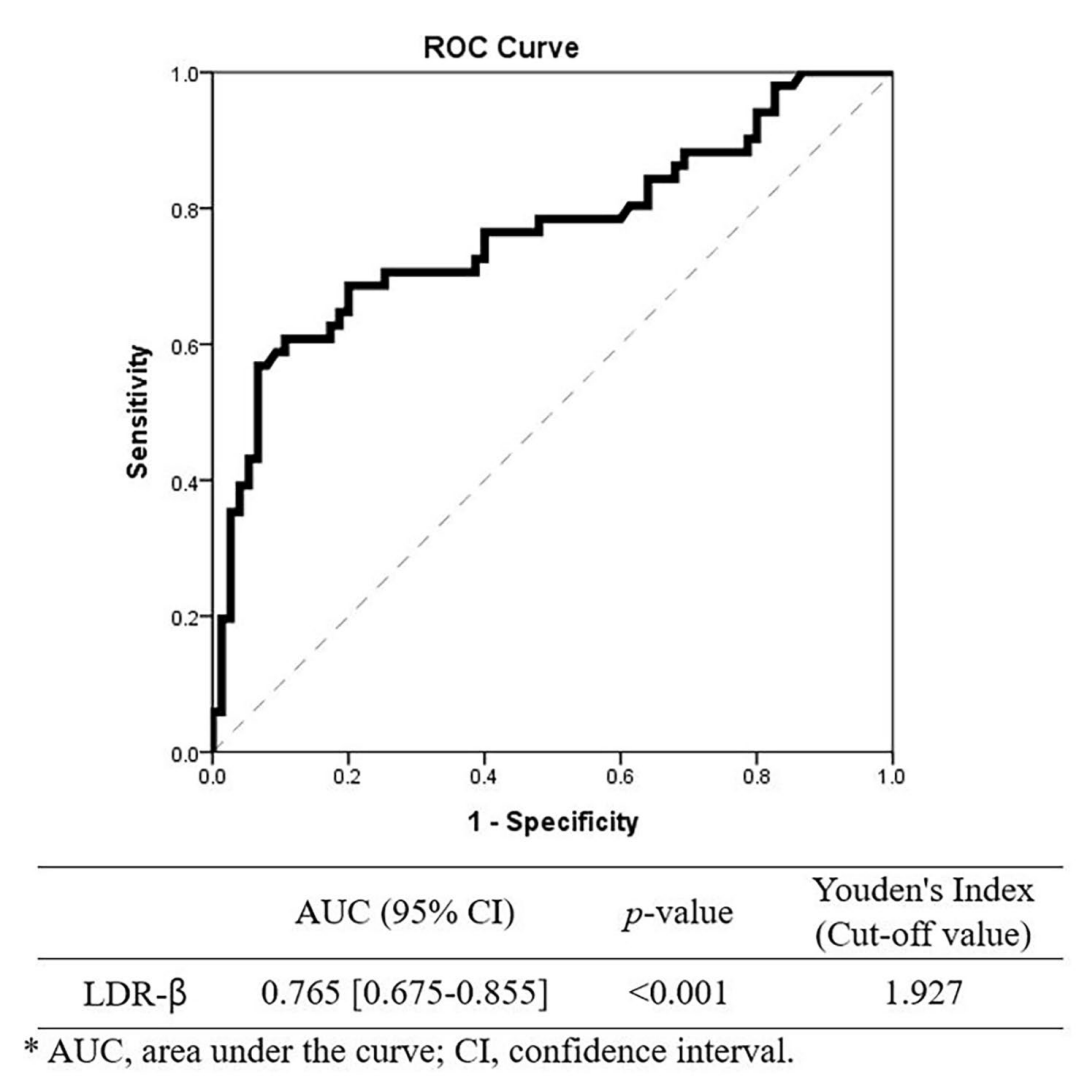
Receiver operating characteristic curve analysis

In the ROC curve analysis, LDR-β had clinically fair discriminatory power between less mismatched tendency and more mismatched tendency in the cohort (AUC = 0.765 [95% CI, 0.675–0.855], *p* < 0.001) (Fig. 5). A threshold LDR-β value of 1.927 would predict severe M-D mismatch with a sensitivity of 56.9% and specificity of 93.3% (*p* < 0.001). For an LDR-β > 1.927, the odds of a severely mismatched femur was 18.455 ([95% CI, 6.374–53.434], *p* < 0.001). The discriminatory power of CFI (AUC = 0.677 [95% CI, 0.581–0.773], *p* < 0.001) was poor. The discriminatory power of LDR-α (AUC = 0.721 [95% CI, 0.625–0.817], *p* < 0.001) and modified MCI (AUC = 0.745 [95% CI, 0.658–0.831], *p* < 0.001), CCR (AUC = 0.700 [95% CI, 0.608–0.791], *p* < 0.001) were fair, but AUC of those indices were all smaller than that of LDR-β.

## Discussion

Preoperative assessment of proximal femur morphology is crucial in femoral stem selection. However, there was a limitation in interpreting the radiograph with the existing radiologic indices. Thus, we suggested novel radiologic indices to detect the delicate differences in the severity of M-D mismatch in the range from LT to 5 cm below the LT. In this study, the evaluation of LDR-β revealed that the point at 3 cm below the LT was an important location for the morphological change in the proximal canal and accordingly, for selecting stem type. To our best knowledge, this is the first study to examine and develop the radiologic indices that could help select the proper stem type in an extreme Champaign-fluted femur. The preoperative evaluation of LDR-β in the simple radiographs with the analyzed cut-off value would help in the assessment of the mismatch in advance and in preparing the appropriate implant.

Preoperatively, surgeons should select a proper type of stem by templating the femur meticulously. In a severe M-D mismatched femur, the type 1 stem may be captured around the narrow distal canal of the femur, not at the intended area of the metaphyseal portion. If neglected, the micromotion of the proximal portion of the stem causes thigh pain [23] and creates a mechanical environment unfavorable for successful osteointegration of the coated portion [6, 24, 25]. Ishii et al. reported that M-D mismatch could cause failed osteointegration, distal hypertrophy, and a lack of proximal spot welds [7]. These problems are potentially avoidable with awareness of the femoral morphology and the functional design of the stem.

All radiographic indices of the bony morphology evaluated in this study showed a statistically significant difference between the two cohorts. Previous indices, however, have been used to describe the general morphology of the femur rather to determine the appropriate stem type in THA. Several authors have tried correlating these indices with a metaphyseal-diaphyseal imbalance of the proximal femur [12, 13, 15, 19]. McGoldrick et al. reported that a higher CFI suggested an M-D mismatched feature [26]. In the midst of the continuous introduction of mid-to short-length femoral stems to the market by several manufacturers [27, 28], these indices were limited in that they reflect too broad range of the proximal femoral canal, which might be irrelevant to the stem insertion. In contrast, LDR-α, which used an internal diameter at 2 cm below the LT, represented a relatively narrow range of the proximal femoral canal. LDR-β, the index with a point 3 cm below the LT, seemed a reliable predictor of severe M-D mismatch.

Preparation of the proximal femur is technically challenging. Thorough knowledge of the implant system and evaluation of the femoral morphology are crucial. In some cases where initial fixation seems achieved but in the distal portion of the type 1 stem in an M-D mismatched femur, a surgeon might perform additional preparation of the femoral canal to avoid the lengthening of the leg, which possibly lead to the periprosthetic femoral fracture [29]. Knowing when to change the stem type from type 1 to type 3 C stem is essential in these situations, and the use of LDR-β is advisable. In addition, if each unique discriminatory cut-off value of LDR-β is presented when developing a new type 1 system, the surgeon can determine the severity of M-D mismatch and select an appropriate system before surgery.

Based on LDR-β and further morphological studies of the interval from LT to point at 3 cm below the LT, it is possible to develop a type 1 stem suitable for the specific ethnic populations by minimizing M-D mismatch. The femoral canal is relatively small in Asian populations [24]. Umer et al. revealed that the femoral canal was narrower in a Pakistani population than in a Western population [30]. Cho et al. reported that women in Korea had relatively small and narrow proximal femurs [31]. Also, most of the commercialized type 1 stems were developed based on the femurs of the Western population with an average canal dimension close to that of Dorr B femurs [32]; therefore severe M-D mismatch occurs more frequently in the Asian population.

Further studies of LDR-β also have advantages on non-Asian populations. Kheir et al. reported a prevalence of Dorr A femur of 21.1% after analyzing patients with a mean age of 71 years [33]. As such, the overall prevalence of Dorr A femur appears lower than that of the Asian, but this should not be overlooked. In addition, Issa et al. revealed that Dorr A femur had a high prevalence of 63.0% in young patients [34], and given that the number of young patients undergoing THA is increasing because of the expansion of indication [35], the morphology of Dorr A femur and M-D mismatch should be further studied and it is helpful to understand the results of this study.

We acknowledged several limitations to this study. First, the study design was limited by the retrospective nature, which might lead to selection bias. Second, since we analyzed the relatively small-sized femurs of female patients, this might be a bias that limited our study. The cut-off value in this study, therefore, needed further validation to be used in femurs with larger sizes and in male patients. However, the proximal femurs of women in Korea tended to be relatively small and narrow; thus, the cut-off value derived from our cohort might be applicable [31, 36]. Third, only one design per type of stem from a single manufacturer was utilized in this study. Further studies with various stems with a similar design should be warranted. Finally, all the indices were measured on plain film radiography. Computed tomography imaging may provide a more detailed three-dimensional assessment of the proximal femoral anatomy [37].

## Conclusions

In conclusion, along with the existing radiologic indices, the index using a ratio based on 3 cm below the LT of the femur seemed crucial in selecting the stem type. Correct assessment of preoperative femoral morphology would be fundamental in the selection of a suitable stem. Since the newly described radiological index, LDR-β, showed the strongest discriminative power in selecting between type 1 and type 3 C stem in severe M-D mismatched femur, we recommend evaluating the index preoperatively for precise planning and thorough preparation.

### Electronic supplementary material

Below is the link to the electronic supplementary material.

Supplmentary Table 1

## Data Availability

The dataset supporting the conclusions of this article is included within the article.

## Abbreviations

THA: Total hip arthroplasty
M-D: Metaphyseo-diaphyseal
CFI: Canal-flare index
CCR: Canal-calcar ratio
MCI: Morphological cortical index
LT: Lesser trochanter
LDR: Lesser trochanter-to-distal ratio
ICC: Intraclass-correlation coefficient
ROC: Receiver operating characteristic
AUC: Area under the receiver operating characteristic curve
