# Supplementary material for: Novel radiologic indices for stem type decision in total hip arthroplasty in patients with metaphyseo-diaphyseal mismatched Dorr A proximal femur
Source: BMC Musculoskelet Disord. 2024 Feb 9;25:124. doi: 10.1186/s12891-024-07223-5 (PMC10854119; doi:10.1186/s12891-024-07223-5)
Supplement: Supplementary file 1 — Supplmentary Table 1 [file 12891_2024_7223_MOESM1_ESM.docx]

**Supplmentary Table 1. Reliability study: ICC values for each index**

|  | ICC of inter-observer reliability | | ICC of intra-observer reliability |
| --- | --- | --- | --- |
| Canal-calcar ratio (CCR) | 0.946 | 0.905 | |
| Canal-flare index (CFI) | 0.901 | 0.912 | |
| Modified morphological cortical index | 0.952 | 0.919 | |
| LT-to-distal ratio-α (LDR-α) | 0.926 | 0.963 | |
| LT-to-distal ratio-β (LDR-β) | 0.954 | 0.928 | |
